# Case series of sebelipase alfa hypersensitivity reactions and successful sebelipase alfa rapid desensitization

**DOI:** 10.1002/jmd2.12066

**Published:** 2019-08-01

**Authors:** Michelle F. Huffaker, Anne Y. Liu, Gregory M. Enns, Suresh Vijay, Antonio J. Amor, N. Franklin Adkinson

**Affiliations:** ^1^ Division of Allergy, Immunology, & Rheumatology, Department of Pediatrics Stanford University Stanford California; ^2^ Allergy & Asthma Medical Group of the Bay Area San Ramon California; ^3^ Birmingham Children's Hospital NHS Foundation Trust Birmingham UK; ^4^ Endocrinology and Nutrition Department, Institut d'Investigacions Biomèdiques August Pi i Sunyer, Hospital Clínic University of Barcelona Barcelona Spain; ^5^ Centro de Investigación Biomédica en Red Fisiopatología de la Obesidad y Nutrición Instituto de Salud Carlos III Spain; ^6^ Johns Hopkins Asthma & Allergy Center Baltimore Maryland

**Keywords:** allergy, anaphylaxis, desensitization, enzyme replacement therapy, hypersensitivity, sebelipase alfa

## Abstract

Allergic immune‐mediated hypersensitivity reactions are known potential complications of enzyme replacement therapy. Sebelipase alfa, recombinant lysosomal acid lipase (LAL), is a potentially life‐altering treatment for patients with LAL deficiency. There is very little information on the diagnosis and management of immediate hypersensitivity reactions to this drug. Here we present three unique cases of hypersensitivity reactions to sebelipase alfa, spanning a broad age spectrum from infancy to adulthood, each managed with successful rapid desensitization.

## 1 INTRODUCTION

Enzyme replacement therapy (ERT) is life‐changing for many with lysosomal storage diseases but treatment can be hindered by immunologic reactions, including infusion and hypersensitivity reactions and development of neutralizing antibodies. Infusion reactions and allergic hypersensitivity reactions can have overlapping features including wheezing, dyspnea, tachycardia, hypotension, and flushing.^1^, ^2^ Both typically occur during administration, and management has previously been described.^1^, ^3^ Classifying a reaction as an infusion reaction or an allergic hypersensitivity is clinically important, as the latter can result in life‐threatening anaphylaxis while the former can be managed with premedication and slowed infusion rate.^1^, ^2^ Here we present three patients with lysosomal acid lipase (LAL) deficiency (OMIM #27800) with hypersensitivity reactions to sebelipase alfa, recombinant LAL, who subsequently underwent successful rapid desensitization. Per study protocol, none of these patients received premedication for initial infusions, but their reactions were refractory to addition of premedications to subsequent infusions.

## 2 CASE 1

A 47‐year‐old Caucasian male with LAL deficiency and cerebral palsy tolerated sebelipase alfa 1 mg/kg every other week without reaction until week 12, when he developed diffuse urticaria, conjunctival injection, rhinorrhea, tachycardia, and tachypnea 90 minutes into infusion. The infusion was stopped and he was given dexchlorpheniramine 5 mg intravenous (IV) and hydrocortisone 100 mg IV with resolution of symptoms within an hour. Two weeks later, after pretreatment with dexchlorpheniramine 5 mg IV and paracetamol 1000 mg IV 15 minutes prior, he again developed diffuse rash, conjunctival injection, rhinorrhea, and tachypnea 88 minutes into the infusion. The same rescue treatment was administered with similar results as the prior infusion. Serum tryptase was 4.7 ng/mL (reference <13.0 ng/mL). Skin prick testing (SPT) and intradermal testing (IDT) to sebelipase alfa were performed per study protocol, with SPT at concentrations of 1:1000 (0.002 mg/mL) and 1:100 (0.02 mg/mL) and IDT at 1:1000. SPT was negative and IDT was positive with development of a 6‐mm wheal. Drug‐specific IgG antibodies were not detectable. Basophil activation test performed by a central lab was positive. Desensitization to sebelipase alfa was recommended based on clinical anaphylaxis and positive skin testing.

Five attempts were made to desensitize the patient to the full dose of 1 mg/kg of sebelipase alfa using a protocol ranging from 9 to 15 steps with initial concentrations as low as 0.001% of the target dose. Before each desensitization, the patient received dexchlorpheniramine 5 mg IV 1 hour prior; the first two attempts also included methylprednisolone 60 mg IV. Within 3‐6 hours of starting each desensitization, the patient developed a diffuse pruritic rash responsive to dexchlorpheniramine 5 mg IV and methylprednisolone 40‐60 mg IV. For the first three desensitizations, the desensitization protocol was then completed without recurrence of symptoms. In the fourth attempt, the patient developed a diffuse pruritic rash and conjunctival injection 70 minutes after initial resolution and the infusion was stopped and methylprednisolone (60 mg IV) was administered. Symptoms resolved within an hour. In the fifth attempt, he had multiple recurrences of diffuse pruritic rash precluding completion, despite dexchlorpheniramine doses. His target dose of sebelipase alfa was reduced to 0.35 mg/kg every other week, which he tolerated by desensitization. Over the following weeks, his desensitization protocol was shortened and simplified without reaction, without dose escalation. The initial tolerated protocol and the final abbreviated protocol are shown (Table 1).

**Table 1:** Sebelipase alfa desensitization protocols for case 1

Step	Solution^a^^,^ b	Rate (mL/h)	Time (min)	Volume infused per step (mL)	Dose administered with this step (mg)	Cumulative dose (mg)
Initial successful desensitization protocol						
1	1	5	15	1.25	0.0030	0.0030
2	1	10	15	2.5	0.0060	0.0090
3	1	20	15	5	0.0120	0.0210
4	2	5	15	1.25	0.0300	0.0510
5	2	10	15	2.5	0.0600	0.1110
6	2	20	15	5	0.1200	0.2310
7	2	40	15	10	0.2400	0.4710
8	3	10	15	2.5	0.5882	1.0592
9	3	20	15	5	1.1765	2.2357
10	3	40	138.75	92.5	21.7643	24.0000
Final densensitization protocol						
1	1	5	15	1.25	0.0300	0.0300
2	1	10	15	2.5	0.0600	0.0900
3	1	20	15	5	0.1200	0.2100
4	1	40	15	10	0.2400	0.4500
5	2	10	15	2.5	0.5888	1.0388
6	2	20	15	5	1.1775	2.2163
7	2	40	15	10	2.3550	4.5713
8	2	80	61.8	82.5	19.4288	24.0000

a Initial protocol: Solution 1:100 mL volume; 0.0024 mg/mL concentration. Solution 2:100 mL volume; 0.024 mg/mL concentration. Solution 3:100 mL volume; 0.23529 mg/mL concentration.

b Final protocol: Solution 1:100 mL volume; 0.024 mg/mL concentration. Solution 2:100 mL volume; 0.2355 mg/mL concentration.

## 3 CASE 2

A 6‐week‐old Asian female with LAL deficiency tolerated her first three infusions of sebelipase alfa at 1 mg/kg every other week. For the fourth infusion, the dose was increased to 3 mg/kg due to poor weight gain and prior experience that infantile‐onset LAL deficiency patients did not respond to doses under 3 mg/kg. One hour after completion of the infusion, she became tachycardic and tachypneic, and vomited once. She recovered without intervention. During the fifth infusion, after administration of 1.6 mL of a 20‐mL dose, she developed facial flushing and tachycardia. The infusion was stopped and she was given chlorphenamine (1.25 mg [250 μg/kg]) intramuscular (IM) and hydrocortisone 25 mg IM with resolution of symptoms. For the sixth infusion, she received premedication with chlorphenamine 1.25 mg IM and hydrocortisone 25 mg IM, and the infusion rate was slowed to start at 0.5 mL/h and increased 0.5 mL/h every 30 minutes. After administration of 6.1 mL, she developed facial flushing, tachycardia, hypoxia, respiratory distress, and stridor. She received epinephrine IM (150 μg of a 1:1000 solution), nebulized epinephrine (1 mL of a 1:10000 solution), hydrocortisone 25 mg IM, and chlorphenamine 1.25 mg IM with resolution of symptoms. SPT 2 weeks later was negative at 1:1000 and 1:100, and IDT was not done. Antidrug antibodies were detected at a titer of 1:96 after the fifth infusion, previously negative. She additionally tested positive for neutralizing antibodies to sebelipase alfa and cell uptake inhibition at titer of 1:500. Preceding baseline serum tryptase levels were within normal limits. Based on the clinical diagnosis of anaphylaxis, desensitization was recommended.

She tolerated desensitization with an 8‐step protocol with 3‐hour intervals, to a target dose of 0.35 mg/kg, without premedication (Table 2). The target dose was subsequently increased. After desensitization to a dose of 1 mg/kg, she developed lip angioedema and erythematous tongue with spontaneous resolution of symptoms. She tolerated desensitization to 2 mg/kg without reaction. Four hours into the subsequent desensitization with a target dose of 3 mg/kg, she developed lip angioedema, irritability, and tachycardia, which resolved when the infusion was stopped. Desensitization to 2 mg/kg was repeated without reaction. She then successfully underwent desensitization to 3 mg/kg without reaction. Over subsequent desensitizations, the infusion rate was increased and desensitization was completed in 4 hours. She continues to tolerate this protocol without reaction.

**Table 2:** Initial sebelipase alfa desensitization protocols for case 2

Step	Solution^a^	Rate (mL/h)	Solution volume infused per step (mL)	Saline volume infused per step (mL)	Dose administered with this step (mg)	Cumulative dose (mg)
1	1	3.33	1.0	9.0	0.02	0.02
2	1	3.33	2.0	8.0	0.04	0.06
3	1	3.33	4.0	6.0	0.08	0.14
4	1	3.33	8.0	2.0	0.16	0.3
5	2	3.33	1.6	8.4	0.32	0.62
6	2	3.33	3.1	6.9	0.62	1.24
7	2	3.33	6.3	3.7	1.3	2.54
8	2	3.33	12.5	7.5	2.5	5.04

a Solution 1: 1000 mL volume; 20 mg/mL concentration. Solution 2: 100 mL volume; 20 mg/mL concentration.

## 4 CASE 3

A 13‐year old Caucasian male with LAL deficiency started on sebelipase alfa at a dose of 1 mg/kg. He developed a pruritic truncal rash 5‐7.5 hours after completion of his first 2‐hour infusion, which was done without pretreatment. The rash resolved with diphenhydramine. He received prednisone 25 mg per os (PO) and cetirizine 10 mg PO premedication for his second infusion, but 8.5 hours post‐infusion he developed oropharyngeal angioedema, dyspnea, difficulty speaking, and a diffuse pruritic rash. Symptoms resolved within an hour of treating with diphenhydramine 25 mg PO. Tryptase was not drawn. SPT and IDT to sebelipase alfa were performed, with SPT at 1:1000 and 1:100 and IDT at 1:1000, 1:100, and 1:10 (0.2 mg/mL). All were negative at the standard reading time of 15 minutes after placement. He then developed a reaction several hours later at the testing site. He had no detectable antidrug antibodies. As his reactions and skin testing conversion occurred several hours after leaving the hospital, he did not have a clinician‐observed reaction, but he and his parents were known to be reliable historians. His history of late‐onset reaction with features consistent with anaphylaxis, worsening upon re‐exposure despite premedication, and possible delayed positive skin testing suggested an immunologically mediated hypersensitivity, so desensitization was recommended.

Initial desensitization was done without premedication to a goal dose of 0.35 mg/kg, with a 10‐step protocol delivered at 2 hours per step (Table 3) and was tolerated well. Because of unknown drug stability at low concentrations, each step used a separate dilution. The goal dose was increased to 1 mg/kg starting with the third desensitization. For each subsequent infusion, the duration and number of steps were gradually reduced to a final protocol with one dilution delivered in six steps, with 12 minutes per step for the first four steps (Table 3). The final desensitization protocol required 138 minutes, still longer than the standard infusion time for his 68‐mg dose (93.75 minutes). After multiple successful desensitization procedures, he continues using this protocol.

**Table 3:** Sebelipase alfa desensitization protocol for case 3

Initial desensitization protocol^a^					
Step (syringe)	Dose (mg)	Volume of normal saline (mL)	Rate (mL/h)	Time (h)	Cumulative dose (mg)
1	0.02	10	5	2	0.02
2	0.04	10	5	2	0.06
3	0.08	10	5	2	0.14
4	0.16	10	5	2	0.3
5	0.31	20	10	2	0.61
6	0.62	10	5	2	1.23
7	1.2	10	5	2	2.43
8	2.5	20	10	2	4.93
9	5	30	15	2	9.93
10	10	50	25	2	19.93

Final desensitization protocol						
Step	Solution^b^	Rate (mL/h)	Time (min)	Volume infused per step (mL)	Dose administered with this step (mg)	Cumulative dose (mg)
1	1	10	12	2	0.544	0.544
2	1	20	12	4	1.088	1.632
3	1	40	12	8	2.176	3.808
4	1	80	12	16	4.352	8.16
5	1	120	30	60	16.32	24.48
6	1	160	60	160	43.52	68

a Initial desensitization protocol: each step is a new syringe.

b Solution 1: 250 mL volume; 0.272 mg/mL concentration; 68 mg total dose in solution.

A summary table of significant data from the three cases is provided (Table 4).

**Table 4:** Summary of cases

Case	Age	Sex	Infusion number when reacted	Skin test results	Other significant testing performed	Time duration of initial desensitization	Time duration of final desensitization
1	47 y	M	7	Positive intradermal	BAT positive	273.75 min	166.8 min
2	6 wk	F	4	Negative skin prick, intradermal not performed	Antidrug antibodies positive Neutralizing antibodies positive	927.6 min	240 min
3	13 y	M	1	Delayed positive	Negative antidrug antibodies	1200 min	138 min

Abbreviation: BAT, basophil activation test.

## 5 DISCUSSION

There are several clinically important lessons from these cases. The first is that clinicians must differentiate typical infusion reactions from hypersensitivity reactions that may require desensitization. Features in these cases suggestive of antigen‐specific, mast cell‐mediated allergic hypersensitivity reactions include urticaria, angioedema, sneezing, pruritus, and positive skin testing. Fever and rigors are more suggestive of typical infusion reactions, while wheezing, dyspnea, tachycardia, hypotension, and flushing are common to both. When available, skin testing can be helpful in determining the diagnosis and appropriate management.^1^, ^2^ Studies have shown that those with infusion reactions with negative skin testing can often tolerate slower infusions without requiring desensitization.^1^ Positive skin testing may confirm immediate allergic hypersensitivity that infusion rate reduction and premedication will not overcome.^1^ The negative predictive value of skin testing to sebelipase alfa is not well established, and a negative skin test is not sufficient to rule out immediate hypersensitivity as demonstrated in the second case. In addition, although features of mast cell degranulation better define hypersensitivity reactions, tryptase elevation is fairly insensitive. Furthermore, hypersensitivity reactions may occur either after multiple uneventful infusions as in cases 1 and 2, or upon first exposure as in case 3.

Clinicians must additionally be aware of the possibility of delayed hypersensitivity reactions. The phenomenon of delayed anaphylaxis suggests either biphasic anaphylaxis with a subtle immediate reaction followed by a more pronounced late phase reaction, or anaphylaxis to a metabolite or by‐product of the substance.^4^ In case 3, the patient had reactions suggestive of either delayed‐onset anaphylaxis or severe delayed infusion reaction. A missed immediate reaction is unlikely as his first infusion was without premedication and was closely monitored, making a missed early phase less plausible. Additionally, his skin testing was initially negative and subsequently may have turned positive. Late‐phase cutaneous reactions have been described in patients with immediate hypersensitivity reactions but they are not well understood.^5^

Delayed‐onset anaphylaxis to an infused medication is not common, and the diagnosis should be approached with caution and if suspected should prompt consultation and skin testing by an allergist. A notable example of delayed anaphylaxis is ingestion allergy to non‐primate mammalian meat via sensitization to galactose‐1,3‐alpha‐galactose (“alpha‐gal”), a carbohydrate moiety.^6^ These patients can have anaphylaxis 4‐6 hours after meat ingestion and first‐dose anaphylaxis to cetuximab, a monoclonal antibody used for gastrointestinal cancers. Sebelipase alfa has a short serum half‐life of only several minutes, making the delayed reaction puzzling in the third case (Kanuma PI^7^). It remains unclear whether the metabolic pathway of ERT therapy with uptake into lysosomes plays a role in the delayed symptoms in case 3. While there is a possibility that this may be a delayed infusion reaction, reports have demonstrated that the use of premedications, including IV steroids, prevent delayed reactions in only 50% of subjects, which raises the possibility that some of these patients had delayed‐anaphylactic or other hypersensitivity reactions.^3^ Perhaps a slow dose escalation protocol akin to a desensitization protocol would benefit patients who react despite premedication and reduced infusion rates.

The delay in skin test results in case 3 without an immediate phase response, in conjunction with the delayed anaphylactic clinical reaction, could be consistent with immune activation by a metabolite of the drug or presentation of the drug only after it is taken up into the lysosome. There are multiple mechanisms for anaphylaxis, the predominant being IgE‐mediated and direct mast cell activation, but IgG‐ and complement‐mediated mechanisms have also been described in animals.^8^ IgG‐mediated anaphylaxis should be distinguished from IgG‐mediated drug neutralization, which reduces drug efficacy rather than causing clinical reactions. Successful treatment of IgG neutralizing antibodies to ERT is described elsewhere.^9^ General binding anti‐sebelipase alfa antibodies as well as specific neutralizing anti‐sebelipase alfa antibodies can be assayed, although in the phase 3 trial of sebelipase alfa, there was no evidence that these antibodies had any clinical significance.^9^, ^10^

The biological mechanism behind desensitization remains unknown, and new desensitization protocols are developed with careful consideration to what has worked historically as well as the novel aspects of the desired drug and the nature of the patient's reaction.^2^, ^11^, ^12^ Each desensitization protocol in this case series was initially developed with prolonged steps. Ultimately, each patient tolerated shorter time intervals on subsequent desensitizations, with individualized protocol adjustments. In cases 2 and 3, desensitization to lower doses preceded successful desensitizations to original or higher doses. We cannot say whether the initial prolonged steps or lower doses were a necessary induction phase, although case 2 suggests so. Desensitization protocols should be created and tailored in consultation with an allergist proficient in recognizing and managing anaphylaxis and differentiating it from infusion reactions.

We believe this is the first reported case series of successful rapid desensitization to sebelipase alfa. In this case series, desensitization was a successful treatment option for patients with both immediate and delayed‐onset reactions suggestive of anaphylaxis and allowed these patients to continue treatment with ERT.

## ANIMAL RIGHTS

This article does not contain any studies with animal subjects performed by any of the authors.

## AUTHOR CONTRIBUTIONS

All authors contributed to the planning, conduct, and reporting of the work described in the article. M.F.H. and A.Y.L. accept full responsibility for the work and/or the conduct of the study, had access to the data, and controlled the decision to publish.

## CONFLICT OF INTEREST

M.F.H., A.Y.L., and A.J.A. declare that they have no conflict of interest. G.M.E. received funding for the conduct of clinical studies from Alexion Pharmaceuticals, Inc. S.V. has been a consultant for Alexion Pharmaceuticals, Inc., received honoraria and travel grants from Alexion Pharmaceuticals, Inc. N.F.A. has been a consultant for Alexion Pharmaceuticals, Inc.

## ETHICAL APPROVAL STATEMENT

For all studies, the protocol, amendments, and subject informed consent were reviewed and approved by an institutional review board (IRB) or independent ethics committee (IEC) prior to initiation of the study. The studies were conducted according Good Clinical Practice (GCP) and International Conference on Harmonisation (ICH) guidelines, applicable government regulations, and Institutional research policies and procedures.

## PATIENT CONSENT

Written informed consent was obtained for all studies.

6

## Data Availability

Qualified academic investigators may request participant‐level, de‐identified clinical data, and supporting documents (statistical analysis plan and protocol) pertaining to this study. Further details regarding data availability, instructions for requesting information and our data disclosure policy will be available on the Alexion.com website (http://alexion.com/responsibility).
